# The Differential Expression of OCT4 Isoforms in Cervical Carcinoma

**DOI:** 10.1371/journal.pone.0118033

**Published:** 2015-03-27

**Authors:** Shao-Wen Li, Xiao-Ling Wu, Chun-Li Dong, Xiu-Ying Xie, Jin-Fang Wu, Xin Zhang

**Affiliations:** 1 Department of Obstetrics and Gynecology, the Second Affiliated Hospital of Medical School, Xi’an Jiaotong University, Xi’an, China; 2 Department of Pediatrics, the Second Affiliated Hospital of Medical School, Xi’an Jiaotong University, Xi’an, China; National Cancer Center, JAPAN

## Abstract

OCT4 is a transcription factor involved in maintaining stem cell phenotype and pluripotential. However, it remains unclear the expression pattern and biological function of OCT4 isoforms in cervical cancer. Here, we reported that both nuclear OCT4A and cytoplasmic OCT4B were overexpressed in CC. OCT4A was responsible for self-renewal of cervical cancer stem–like cells (CCSCs). Furthermore, OCT4B overexpression in SiHa cervical cancer cell line significantly increased cell proliferation and tumorigenesis by inhibiting apoptosis. Moreover, OCT4B enhanced angiogenesis by the upregulation of CD34, VEGF, HIF-1α and IL-6, and promoted tumor cell mobility to the surrounding tissue by the upregulation of MMP2 and MMP9, and the induction of epithelial-mesenchymal transition (EMT). In conclusion, nuclear OCT4A may serve as a marker of CCSCs and the driving force for cervical cancer metastasis and recurrence, while cytoplasmic OCT4B may cooperate with OCT4A to regulate the progression of cervical cancer through inducing angiogenesis and EMT.

## Introduction

Cervical cancer (CC) is the third most common cancer among women worldwide [1]. Epidemiological studies have suggested that multiple risk factors are involved in cervical carcinogenesis, including human papillomavirus (HPV) infection, smoking, and sexual behavior[2]. Widespread cervical screening tests such as Pap smear and HPV DNA testing at an early stage and treatment of precancerous cervical lesions have dramatically reduced the incidence of invasive CC [3]. However, genetic and molecular events contributing to the initiation and progression of CC have not yet been fully understood. Recently, cancer stem cells (CSCs) including cervical cancer stem cells have become a topic of intensive investigations[4,5]. Notably, aberrant expression of certain stem cell-related nuclear transcription factors, such as OCT4 [6,7], SOX2 [8] and NANOG [9], could contribute to cervical carcinogenesis. However, the molecular mechanisms by which these factors promote cervical carcinogenesis have not been fully explored.

*Oct4* (Oct3/4 or POU5F1), a member of the POU family of transcription factors, plays a pivotal role in the maintenance of self-renewal and pluripotency in embryonic stem cells (ESCs) [10]. The human Oct4 gene, located on chromosome 6, consists of five exons and can be alternatively spliced into three main isoforms OCT4A [11], OCT4B [11] and OCT4B1 [12], and generate four proteins OCT4A, OCT4B-190, OCT4B-265, and OCT4B-164. Oct4A and Oct4B/B1 are both functionally and structurally divided into an N-terminal transcriptional activation domain, a central POU domain and a cell type-specific transactivation domain at the C-terminus [13]. OCT4A, generally refered as OCT4, is specifically expressed in the nucleus of ESCs and regulates the stemness of pluripotent cells [14,15]. However, accumulating reports have raised questions about OCT4 as a pluripotency marker because OCT4 is also expressed in human somatic tumor tissues and cells [16,17,18], which may arise from pseudogene transcripts, protein isoforms and DNA contamination [19,20,21,22]. The localization of the different OCT4 isoforms may be correlated with their diverse functions. Compared to OCT4A, OCTB is mainly located in the cytoplasm [14,23]. Cauffman et al. reported different spatial expression patterns of OCT4A and OCT4B during human embryogenesis, suggesting that OCT4A but not OCT4B was responsible for the stemness properties [23,24]. However, Mueller et al. demonstrated that OCT4B or other splice variants instead of OCT4A was present in 42 somatic tumor cell lines [25]. Although the protein product of OCTB1 has not yet been identified, OCT4B1 isoform has been considered as a putative marker for stemness[12,26]. Taken together, considering the complexity and variety of OCT4 spliced variants and protein isoforms, in this study we aimed to investiagte the expression pattern and biological function of OCT4A and OCT4B OCT4 isoforms in cervical cancer.

## Materials and Methods

### Cell culture

Human cervical cell lines (HeLa, SiHa, and C-33 A) and carcinoma cell line Tera-1 were purchased from the American Type Culture Collection (ATCC) and maintained in Dubelcco’s modified Eagle’s medium (DMEM-HIGH Glucose; Sigma-Aldrich, St Louis, MO) supplemented with 10% fetal bovine serum (FBS, Invitrogen, Carlsbad, CA) and 1% penicillin-streptomycin (Sigma) at 37°C, 5% CO2 air atmosphere.

### Isolation and culture of CCSCs

For tumorspheres culture, tissue samples were obtained from the Department of Gynecology and Obstetrics, Second Affiliated Hospital of Xi'an Jiaotong University Medical College (Xi’an, China). Briefly, cells were seeded at a density of 1,000 cells/well in 6-well, ultra low attachment plates (Corning, NY) and maintained in DMEM/F12 medium (Sigma) supplemented with B27 (Invitrogen) mixed with 20 ng/ml epidermal growth factor (EGF, Invitrogen), 20 ng/ml basic fibroblast growth factor (bFGF, Invitrogen) for 2 weeks. For serial tumorsphere formation assays, spheres were passaged by digestion with 0.05% trypsin/EDTA and sieved through a cell strainer with 40-μm nylon mesh to achieve a single-cell suspension and then re-plated in complete fresh medium as described above. The formed tumorspheres were examined and counted under a microscope. Sphere-forming efficiency (SFE) was calculated from first through the fifth generation (G1-G5) using the formula: (number of spheres/number of cells plated) × 100. All experiments were done in triplicate. To induce differentiation, tumorspheres were plated onto glass cover slips pre-coated with poly-L-lysine (Sigma) with FBS-supplemented medium every 2 days (total 7 days).

For separation of the aldehyde dehydrogenase (ALDH)-positive population, Aldefluor analysis was performed using the Aldefluor kit (Stem Cell Technologies, Vancouver, BC, Canada), according to the manufacturer’s instructions. Briefly, trypsinized cells were suspended in ALDEFLUOR assay buffer containing ALDH substrate, Bodipy-aminoacetaldehyde (BAAA), in the absence or in presence of 5 μl of ALDH inhibitor diethylaminobenzaldehyde (DEAB 1.5 mM in 95% ethanol stock solution), and incubated at 37°C for 60 min. Subsequently, cell pellets were resuspended in Aldefluor detection buffer and ALDH1 expression was evaluated by flow cytometry using a fluorescence-activated cell sorter (FACS) ARIA (Becton Dickinson, Franklin Lakes, NJ). The data were analyzed using FlowJo software (Tree Star Inc., Ashland, USA).

### Plasmids, siRNA and transfection

To construct pIRES2-EGFP-OCT4A and pIRES2-EGFP-OCT4B, hTera-1 cell-derived cDNAs were amplified using the following primers: OCT4A-F (5’-GATCGGATCCATGGCGGGACACCTGGCT-3’) and OCT4A-R (5’_-GATCACCGGTGCTCCGTTTGAATGCATGGG-3’); OCT4B-F (5’- GATCGGATCCATGCACTTCTACAGACTATTCCTTGGGGCC-3’) and OCT4B-R (5′ - GATCACCGGTTCAGTTTGAATGCATGGG-3′). The products were digested with BamHI and AgeI and subcloned into pIRES2-EGFP to generate pIRES2-EGFP-OCT4A and pIRES2-EGFP-OCT4B vectors. All constructs were verified by sequencing. Transfection was performed with Lipofectamine 2000 (Invitrogen) according to the manufacturer’s instructions, and stable clones from SiHa cells were selected with 1 mg/ml G418 (Calbiochem, La Jolla, CA).

To knockdown OCT4B, SiHa cells were transfected with OCT4B and scrambled control siRNA (GeneChem, Shanghai, China) for 48 h using Lipofectamin2000 (Invitrogen).

### RT- PCR and quantitative real-time PCR (qRT-PCR)

Total RNA was extracted from cultured cells with TRIzol reagent (Invitrogen) and digested with RNase-free DNase-I (Invitrogen) to remove any DNA contamination. Reverse transcription was performed using RevertAid First Strand cDNA Synthesis Kit (Fermentas, Burlington, Ontario, Canada). Human OCT4A and OCT4B were amplified with the specific primers (Table 1). PCR products were separated on a 2% agarose gel, photographed with The Molecular Imager Gel Doc XR+ system (Bio-Rad, Hercules, CA), and quantified using the ImageJ program (NIH Image, Bethesda, MD) by normalization to glyceraldehyde 3-phosphate dehydrogenase (GAPDH). qRT-PCR was performed with an IQ5 Real-Time PCR Detection System (Bio-Rad) with SYBR Premix Ex Taq II (TaKaRa) according to the manufacturer’s instructions. Quantitation of the relative gene expression levels was normalized to GAPDH using the^ΔΔ^ *C*_*T*_ method of quantitation. All experiments had at least biological duplicates and assay triplicates.

**Table 1:** Primers for RT-PCR and qRT-PCR.

gene	sequence (5'-3')	product size (bp)
OCT4A	CGTGAAGCTGGAGAAGGAGAAGCTG	247
	CAAGGGCCGCAGCTTACACATGTTC	
OCT4B	ATGCATGAGTCAGTGAACAG	303
	CCACATCGGCCTGTGTATAT	
Sox2	CGCCCCCAGCAGACTTCACA	170
	CTCCTCTTTTGCACCCCTCCCATTT	
Nanog	AGTCCCAAAGGCAAACAACCCACTTC	164
	ATCTGCTGGAGGCTGAGGTATTTCTGTCTC	
BMI-1	TAAGCATTGGGCCATAG	140
	ATTCTTTCCGTTGGTTGA	
Klf4	GAAATTCGCCCGCTCAGATGAACT	125
	TCTTCATGTGTAAGGCGAGGTGGT	
GAPDH	GAAGGTGAAGGTCGGAGTC	226
	GAAGATGGTGATGGGATTTC	

### Immunostaining

A total of 50 CC samples were obtained by surgery from patients who had no previous chemotherapy, immunotherapy, or radio-therapy and visited the Second Affiliated Hospital of Xi’an Jiaotong University Medical College (Xi’an, China) between January 2010 and December 2013. The study protocol was approved by the ethical committee of the Second Affiliated Hospital of Xi’an Jiaotong University Medical College. All patients provided written informed consent before specimen collection.

Sections from the paraffin-embedded CC tissues and tumorspheres were deparaffinized in xylene and rehydrated in graded alcohol. After incubation in citrate buffer (10 mM, PH6.0), the sections were stained with primary antibodies for OCT4 (SC-8269, SC-5279, Santa Cruz Biotechnology, Santa Cruz, CA) and CD34 (sc-19621, Santa Cruz Biotechnology). Nuclei were counterstained with hematoxylin and coverslipped. All slides were examined under an Olympus-CX31 microscope (Olympus, Tokyo, Japan).

### Western blot analysis

Protein concentration of the lysates of cells and tumor tissues were measured using the Bradford assay. Equal amounts of protein extracts were separated by 10% sodium dodecyl sulfate polyacrylamide gel electrophoresis (SDS-PAGE) gels and transferred to ployvinylidene fluoride (PVDF) membranes (Milipore, Billerica, MA). The membranes were incubated with primary antibodies for β-actin (sc-47778), human E-Cadherin (sc-8426), N-Cadherin (sc-7939), matrix metalloproteinase (MMP) 2 (sc-10736), MMP9 (sc-21733), Snail 1 (sc-28199), Twist (sc-15393), VEGF (sc-7269), and IL-6 (sc-1265) (Santa Cruz Biotechnology, Santa Cruz, CA, USA), Slug (Cat. #9585) and HIF-1α (Cat. #3716) (Cell Signaling Technology, Danvers, MA, USA). Blots were visualized with a secondary antibody coupled to horseradish peroxidase (Santa Cruz Biotechnology) and an ECL detection system (Millipore).

### Cell proliferation and cell viability assays

For cell growth, the cells (1×10^5^) were seeded in triplicate in 35-mm tissue culture dishes and cultured for 7 days; the cells were harvested and counted every other day using a hemocytometer under light microscopy. A cell growth curve was made to assess cell proliferation. For cell viability assay, cells (1×10^3^) were seeded in 96-well plates and assessed every other day (7 days total) using 3-(4, 5-Dimethyl-1, 3-thiazol-2-yl)-2, 5-diphenyl-2H-tetrazol-3-ium bromide (MTT, Sigma) dye according to standard protocols. Briefly, 20 μl MTT (5 mg/ml) was added into each well and incubated for 4 h at 37°C, and then 150 μl of dimethyl sulfoxide dissolving (DMSO) was added into each well. The optical density was measured at 490 nm. Three independent experiments were performed. For colony formation assay, 200 cells were cultured in triplicate in 10-cm dishes and exposed to fresh media (with 10% FBS) every 3 days for 2 weeks. Then the cell colonies with more than 50 cells were stained with Giemsa (Sigma) and counted using a dissecting microscope to determinate colony formation efficiency (CFE). Each experiment was performed in triplicate.

### Growth inhibition by chemotherapeutics *in vitro*

Cells were seeded in 96-well plates and cultured for 24 h without treatment and then incubated with various concentrations of cisplatin (0, 0.5, 1, 2.5, 5, or 10 μg/mL; Sigma). After 72 h the MTT test was performed to determine cell viability. Three independent experiments were performed.

### Flow cytometry analysis

For cell cycle analysis, the cells were harvested and fixed with ice-cold ethanol overnight at 4°C. After washing twice with PBS, the cells (10^6^ cells/tube) were treated with RNase A and stained with propidium iodide (PI) (Sigma) in the dark, and then analyzed by flow cytometry (FACScan; BD). In addition, the cultured cells (10^5^ cells /tube) were harvested and stained in duplicate with APC annexin V and PI (BD) to characterize spontaneous cell apoptosis according to the manufacturer’s instructions. Each experiment was performed in triplicate.

### Xenograft tumorigenicity assay

The animal experiments were performed in accordance with the institutional guidelines for use of laboratory animals. BALB/c athymic nude mice (4–6 weeks) were injected subcutaneously with SiHa-OCT4A or SiHa-OCT4B and SiHa-GFP cells into the right and left side, respectively, and housed in a pathogen-free facility. Tumors were measured with calipers at daily intervals after injection as indicated and the volume was calculated using the following formula: (length × width^2^)/2. At the end of experiments, subcutaneous tumors were surgically excised, weighed, and photographed. The experimental protocols were evaluated and approved by the Animal Care and Use Committee of the Medical School of Xi’an Jiaotong University.

### Statistical analysis

Statistical analyses were performed using GraphPad Prism 5.01 software (GraphPad Software, La Jolla, CA). All data were presented as mean ± standard deviation (SD). Univariate analysis was performed using Student's t test or a one-way analysis of variance (ANOVA) test. Difference between groups was determined using two-way ANOVA test. *P* < 0.05 was considered to be statistically significant.

## Results

### Identification of OCT4 isoforms in human CC

**Fig 1:**
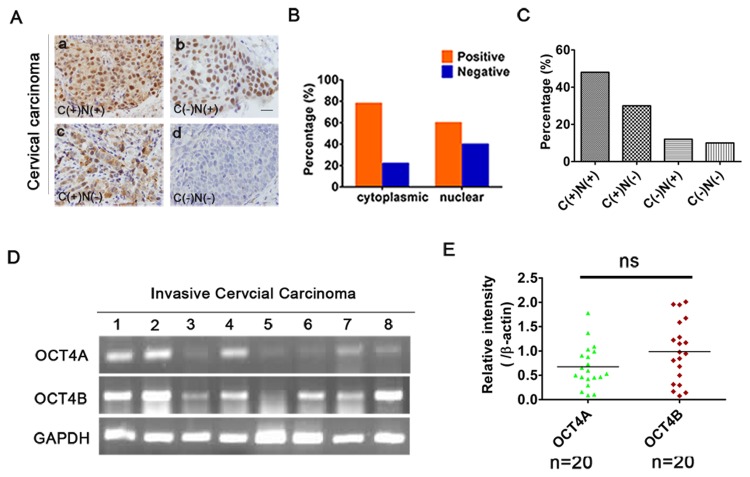
Identification of OCT4 isoforms in human cervical cancer. (A), OCT4 expression status in 50 cervical cancer patients was divided into four groups based on staining patterns and subcellular localization: cytoplasmic and nuclear (C+/N+); cytoplasm (C+/N-); nuclear positive (C-/N+); and complete loss of staining (C-/N-). Scale bars, 50 μm. Magnification, ×100. (B), The positive percentage of cytoplasmic and nuclear staining of OCT4. (C), The positive rates of (A). (D), RT-PCR analysis of OCT4A and OCT4B mRNA levels in 20 CC. (E), Scatter plot showing the relative mRNA levels of OCT4A and OCT4B. (*t*-test, *P* > 0.05). Data shown were the mean ± SD. ns: no significance.

OCT4 is known to be a critical pluripotency marker and has a nuclear localization. First we examined OCT4 expression in 50 CC samples by IHC using polyclonal antibody SC-8629. Unexpectedly, OCT4 protein was not only localized in the nucleus, but also in the cytosol (Fig. 1A). The positive rates of OCT4 staining were 60% (30/50; nuclear) and 78% (39/50; cytoplasmic), respectively (Fig. 1B). Furthermore, OCT4 expression patterns were divided into four groups based on staining intensity and subcellular localization: cytoplasmic and nuclear (C+/N+); cytoplasm (C+/N-); nuclear positive (C-/N+); and complete loss of staining (C-/N-). Therein, each positive percentage of OCT4 staining was 48% (24/50), 30% (15/50), 12% (6/50), and complete loss of OCT4 staining occurred in a minor subset of human tumors (10%/5/50) (Fig. 1C), which predicted that OCT4 expression is positively correlated with cervical carcinogenesis. Furthermore, to clarify the subcellular distribution of OCT4 isoform in CC, OCT4 mRNA level were measured in 20 tumor tissues using RT-PCR (Table 1, Fig. 1D). Similar to the results of IHC, OCT4B transcription was slightly higher than that of OCT4A, but the difference was not significant (Fig. 1E). These results showed that both OCT4A and OCT4B were highly expressed with different subcellular localization in CC at similar levels.

### Identification of OCT4 isoforms in CCSCs

**Fig 2:**
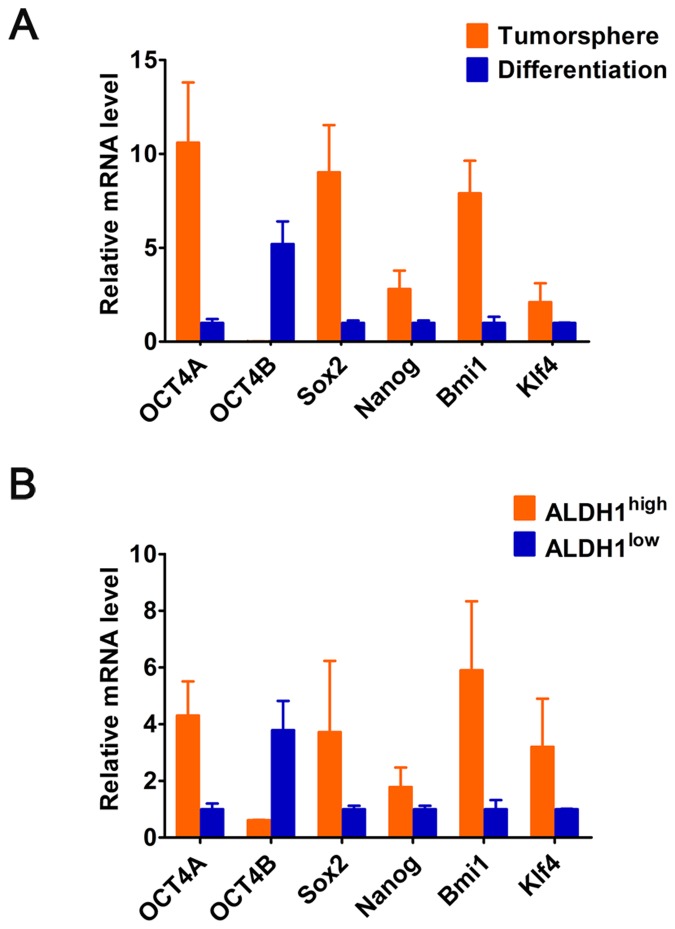
The relative mRNA levels of OCTB and the stem cell-related genes (OCT4A, Sox2, Nanog, Bmi1 and Klf4) in (A) the tumorsphere and differentiated tumorshperes, and (B) ALDH^high^ and ALDH^low^ cells from primary tumors. GAPDH served as a loading control.

We isolated sphere-forming cells (i.e. CCSCs) from primary CC and cultured in suspension at low density in serum-free sphere medium (S1a Fig.). qRT-PCR showed that expression levels of stem markers (OCT4, Sox2 and Nanog Bmi-1, and Klf4) were high in tumorspheres, but then downregulated under differentiation conditions, which verified the stemness signature of tumorspheres formed (Fig. 2A). However, OCT4 was specifically expressed in the nucleus (*white arrow*) and cytoplasm (*red arrowhead*) of tumorspheres (S1b and c Fig.), presumably due to a novel OCT4 alternatviely-spliced variant (i.e. *OCT4B*1). Unlike OCT4A, OCT4B was mainly detected in differentiated tumorsphere cells, but not in tumorshpere (Fig. 2A). Furthermore, ALDH enzymatic activity validated that ALDH1^high^ cells expressed increased levels of OCT4A and other stem-cell-related genes, while ALDH1^low^ cells expressed significantly elevated level of OCT4B (Fig. 2B). Overall, these results suggest that OCT4A but not OCT4B is responsible for maintenance of the stemness properties of CCSCs.

### The expression patterns and role of OCT4A and OCT4B in cervical cancer cell lines

**Fig 3:**
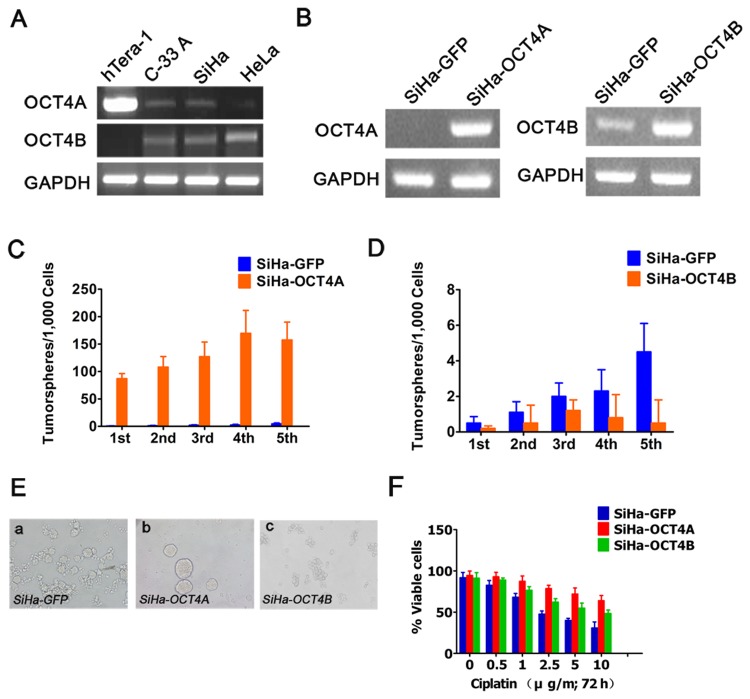
Effects of OCT4A and OCT4B on self-renewal in cervical cancer cell lines. (A), RT-PCR analysis of OCT4A and OCT4B expression in the indicated cell lines. Tera-1 cells as a positive control. (B), Identification of the stable transfected cells. GAPDH served as the loading control. (C) and (D), The number of tumorspheres/1,000 cells was counted from 5 consecutive passages. (E), Representative images of tumorspheres formed from the indicated cells. (F), The chemosensitivity of OCT4A- and OCT4B-overexpressing cells to cisplatin for 72 h.

RT-PCR analysis showed that compared with Tera-1 cells, OCT4A transcript was weakly detected in C-33A and SiHa cells, and could not be detected in HeLa cells (Fig. 3A). However, OCT4B transcripts were detectable in three cell lines, but not in Tera-1 cells. To understand the biological functions of the two *OCT4* isoforms in cervical cancer cells, we established stable SiHa-OCT4A, SiHa-OCT4B and SiHa-GFP control cell lines (Fig. 3B). Sphere formation efficiency (SFE) in the first generation was 8.7± 0.92% for SiHa-OCT4A, 0.05± 0.036% for SiHa-OCT4B, and 0.02± 0.013% for SiHa-GFP, respectively (Fig. 3C and D). Upon serial passage from G1-G5, SFE was gradually increased in SiHa-OCT4A cells, indicating an increased sphere formation capacity. Conversely, SiHa-OCT4B generated few or even no tumorspheres during passages. For morphologic phenotypes, OCT4A-expressing cells displayed the typical nonadherent sphere, while OCT4B-expressing cells exhibited only loose cell aggregates (Fig. 3E). Furthermore, similar to OCT4A, OCT4B-overexpressing cells displayed the stronger resistance to cisplatin (Fig. 3F). Taken together, these results further confirmed that OCT4A promoted tumorshpere formation in cervical cancer cell lines and increased the resistance of tumor cells to cytotoxic drugs.

### OCT4B promotes cervical cancer cell proliferation and tumorigenesis

**Fig 4:**
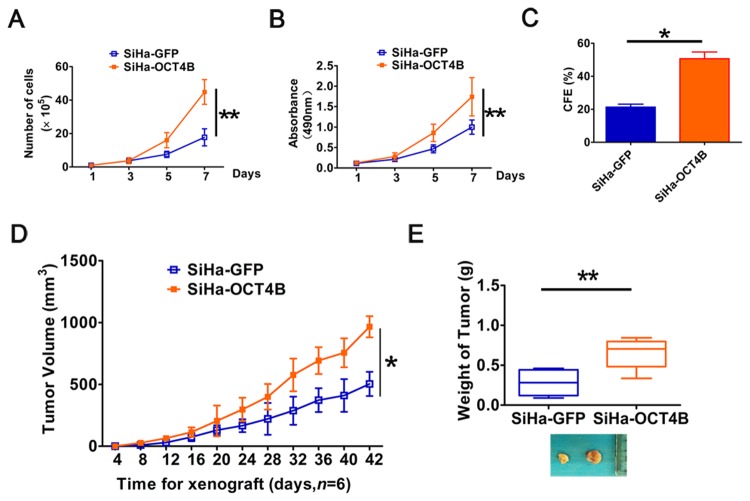
Ectopic OCT4B expression promotes cervical cancer cell proliferation and tumorigenesis. (A), The growth curves and (B) MTT assay to examine the proliferation of SiHa cells. (C), Colony formation assay to evaluate colony-forming ability of OCT4B-overexpressing cells. (D), Tumor growth curves were calculated based on monitoring performed every 4 days post-transplant. (E), The xenograft tumors were dissociated and weighed. The data were shown as mean ± SD (A,B and D were determined by Two-way ANOVA test, while C and E were determined by Student’s *t-*test).*, *P*<0.05; **, *P*<0.01.

To further explore the role of OCT4 in the tumorigenesis of cervical cancer, we examined the effects of OCT4B on cervical cancer cell proliferation *in vitro* and tumorigenesis *in vivo*. Cell growth curves (Fig. 4A) and MTT assays (Fig. 4B) revealed that ectopic expression of OCT4B significantly increased cervical cancer cell growth. Similarly, the numbers of colonies formed in SiHa-OCT4B were dramatically larger than in SiHa-GFP control cells (*P*<0.01, Fig. 4C). Furthermore, in xenograft assay the growth of solid tumors in the SiHa-OCT4B group was significantly increased compared to control cells (*P*<0.01, Fig. 4D). The average volume (966.5 ± 85.56 mm^3^) and weight (0.65 ± 0.19 g) of xenograft derived from SiHa-OCT4A cells at 42 days after implantation were significantly more than the volume (504± 97.581 mm^3^) and weight (0.28 ± 0.15 g) of xeografts derived from SiHa-GFP cells, respectively (*P*<0.01, Fig. 4E). Collectively, these data suggest that OCT4B enhances the proliferation and tumor formation in cervical cancer cells.

### OCT4B inhibits the apoptosis of cervical cancer cells

**Fig 5:**
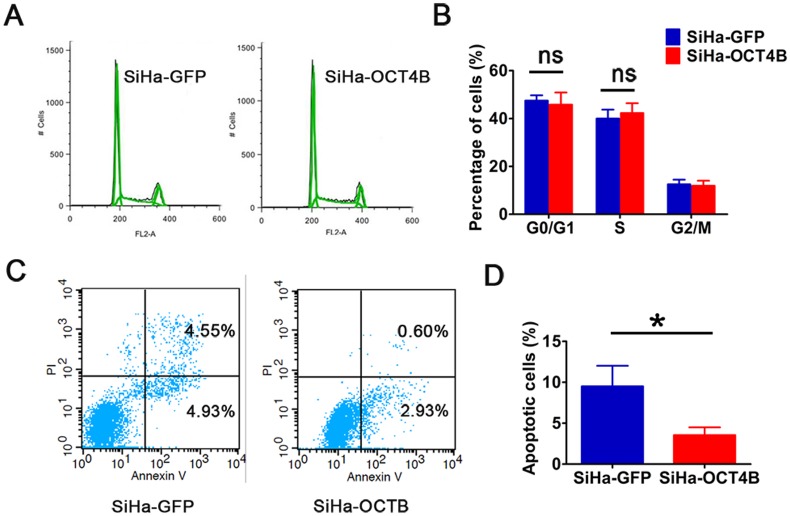
OCT4B promotes cell growth through inhibiting apoptosis. **(A)** Cell cycle analysis of SiHa-GFP and SiHa-OCT4B cells. (B). The distribution of the different phases is indicated. Data were presented as the mean (SD) of triplicates. ns: no significance. (C) Flow cytometry analysis of apoptotic SiHa-GFP and SiHa-OCT4B cells. (D) The percentage of apoptotic cells (n = 3, *t*-test, *P* < 0.05).

To understand how OCT4B promotes cervical cancer cell proliferation and tumorigenesis, we detected the cell cycle and apoptosis. We observed that there was no statistical significance in the proportions of cells in G0/G1 Phase and S phase between SiHa-OCT4B and SiHa-GFP cells (Fig. 5A and B), suggesting that OCT4B has no significant effect on cell cycle. However, OCT4B significantly decreased the ratio of apoptotic cells (Fig. 5C and D). These results suggest that OCT4B promotes the proliferation and tumorigenicity of CC cells through its anti-apoptosis activity.

### OCT4B enhances cervical cancer angiogenesis and EMT

**Fig 6:**
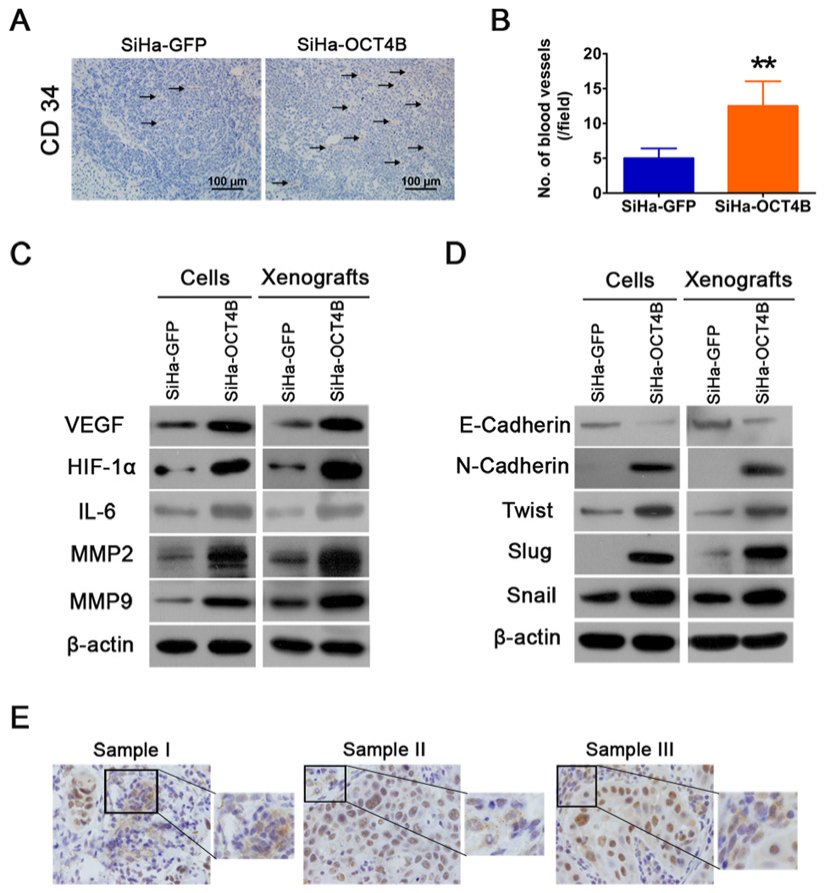
OCT4B promotes cervical cancer angiogenesis and EMT. (A). Tumor xenografts were stained with anti-CD34 antibody. (B). The number of blood vessels was counted. Each column represented the mean ± SD. **, *P*<0.01. (C). Western blot analysis of angiogenesis-associated proteins VEGF, HIF-1α and IL-6, and MMP2 and MMP9. (D) Western blot analysis of EMT-associated proteins E-cadherin, N-cadherin, Twist, Slug and Snail. β-actin was used as loading control. (E) Cytoplasmic OCT4 expression with mesenchymal features in cervical cancer. Three representative samples were shown.

Angiogenesis is a critical step in solid tumor progression and an effective blood supply is a prerequisite of tumor growth, local expansion and metastasis [27]. We thus examined the expression of CD34, a specific endothelial cell marker, in sections from tumor xenografts, and found that there was a clearly increased microvascular density (MVD) in tumors overexpressing OCT4B (Fig. 6A and B). We next assessed the effects of OCT4B on the tumor microenvironment by measuring markers of angiogenesis in SiHa-OCT4B and SiHa-GFP cells and xenografts. Western blot analysis showed that the expression of VEGF, HIF-1α and IL-6 increased in SiHa-OCT4B groups (Fig. 6C). Moreover, OCT4B upregulated the expression of MMP2 and MMP9, which are known to promote tumor invasion.

EMT are implicated in conversion of early-stage tumors into invasive malignancies [30]. We therefore detected the markers of EMT such as E-cadherin, N-cadherin,Twist, Slug, and Snail in OCT4B-expressing cells and xenografts (Fig. 6D). However, in some cases of OCT4 staining in CC, we observed that cytoplasmic OCT4 expression, with mesenchymal cell features, appeared in stromal cells surrounding the cancer nests of nuclear OCT4 expression (Fig. 6E). Taken together, our results demonstrate that OCT4B promotes cervical cancer angiogenesis and EMT.

## Discussion

It is well known that human *OCT4* gene functions as a master regulator for the pluripotency and self-renewal of ESCs. Recent investigations indicate that Oct4 is detected in some somatic tumors such as hepatoma [28] and breast cancer [29]. In addition, the expression level of OCT4 in HPV16-positive cervical cancer cells (HeLa and Caski) was higher than that in HPV-negative cervical cancer cells (C-33A) [7]. In our study, IHC analysis of 50 CC tissues showed that positive OCT4 staining was not only localized in the nucleus, but also in the cytoplasm, which is different from the previous description about OCT4 as a nuclear protein. OCT4 encodes two chief isoforms that are generated by alternative splicing, OCT4A and OCT4B, composed of 360 and 265 amino acids, respectively. Both OCT4A and OCT4B have an N-terminal transcriptional activation domain, a central POU domain, and a C-terminal cell type-specific transactivation domain, they have identical POU DNA-binding and C-terminal transactivation domains but different N-terminus [12,20]. It is suggested that OCT4A and OCT4B can be distinguished by their distinct subcellular localization [30]. To characterize OCT4 isoform and confirm their specificity and cellular localization, the polyclonal antibody SC-8629 against the C-terminus of OCT4 and mouse monoclonal antibody (SC-5279) against the N-terminus of OCT4 were used to detect nuclear OCT4A in 10 cervical cancer samples. As show in S2 Fig., both OCT4 antibodies revealed nuclear localization of OCT4 in positive control cells Tera-1[31]. In cervical cancer samples, nuclear staining of OCT4 was detected by SC-5279 but both nuclear and cytoplasmic staining of OCT4 were detected by SC-8629, further indicating the presence of alternative OCT4 isoforms in cervical cancer samples [32].

Next, we isolated CCSCs from the primary tumor cells and assessed the potential function of OCT4A and OCT4B in cervical cancer initiation. We found that OCT4A was mainly expressed in tumorsphere or ALDH^high^ (i.e. CCSCs), while OCT4B was mainly expressed in differentiated spheres or non-CCSCs, suggesting that OCT4A but not OCT4B serves as a putative marker of CCSCs. In addition, OCT4A overexpression in SiHa cells produced typical nonadherent spherical clusters in serum-free medium, with similar morphologic phenotypes to primary tumorspheres. Moreover, SFE of OCT4A-expressing cells was gradually increased from the first to the fifth generation (G1-G5). In agreement with our results, Kim et al. showed that OCT4 increased the number of breast cancer stem cells [33]. However, OCT4B-overexpressing cells only generated some loose cell aggregates. These findings suggest that OCT4A, as a pluripotency marker, is responsible for maintaining self-renewal of CCSCs, and contributes to the initiation of cervical carcinoma. It is widely believed that the self-renewing cell populations might evade chemotherapy and eventually lead to tumor recurrence. Similar to OCT4A, OCT-4B-expressing cells exhibited less sensitivity to chemotherapy, consistent with previous report [34].

Several studies have investigated the function of OCT4 in carcinogenesis. OCT4A promoted tumorigenesis by inhibiting apoptosis through microRNA125b/BAK1 pathway [35]. Monsef et al. reported that cytoplasmic isoform 2 of OCT4 was present in prostate cancer and benign prostate hyperplasia [36]. OCT4B might represent a clinical prognostic biomarker for prostate cancer patients [30]. OCT4B-190 has been reported to inhibit cell apoptosis induced by heat shock [37], while OCT4B-265 promoted cell apoptosis under genotoxic conditions [38]. However, few studies have explored the role of OCT4B in CC progression. In this study, by ectopic expression of OCT4B in SiHa cells we demonstrated that OCT4B promoted cervical cancer cell proliferation and xenograft growth, and inhibited cell apoptosis. To further confirm the oncogenic role of OCT4B in cervical carcinogenesis, we employed RNA interference to knockdown OCT4B in SiHa cells (S3 Fig.). Our results demonstrated that silencing of OCT4B in SiHa cells significantly inhibited cell growth and increased cell apoptosis. Taken together, these data cleraly suggest that OCT4B plays oncogenic role in cervical cancer due to its anti-apoptotic activity.

Tumor growth and metastasis depend on the development of a neovasculature in and around the tumor [27,39]. Angiogenesis is regulated by the balance between stimulatory factors released by the tumor and its environment [40]. In this study, we found the upregulation of VEGF, HIF-1α, IL-6, MMP-2 and MMP-9 in tumor xenografts derived from OCT4B-expressing cells, suggesting that OCT4B increases endothelial cell migration, vascular sprouting *in vitro*, and vasculature formation in cervical cancer to promote tumor growth, relapse and metastasis. EMT is one of the hallmarks of malignant transformation [41]. Therefore, we wondered whether OCT4B may also induce EMT to promote CC metastasis. Ectopic expression of OCT4B resulted in the inhibition of E-cadherin and the induction of N-cadherin. In addition, the expression of Twist, Snail and Slug was increased in OCT4B-expressing cells and xenografts. These findings suggest that OCT4B enhanced EMT of cervical cancer cells. However, since OCT4A and OCT4B are the protein products of alternative transcriptions of the same *OCT4* gene, it is possible that cancer cells may alternate the expression of the two isoforms to maintain a balance between a metastatic and a static phenotype. Further studies will be needed to test this interesting hypothesis.

In summary, we have shown that both OCT4A and OCT4B are highly expressed in CC at different subcellular locations. OCT4A appears to be responsible for stemness of CCSCs and triggers cervical carcinogenesis, while OCT4B promotes cervical tumor growth by the regulation of apoptosis, angiogenesis and EMT. Therefore, our findings suggest that cytoplasmic OCT4B may act co-operatively with OCT4A to regulate the progression of CC and provide new insight into the function of OCT4 isoforms in carcinogenesis. Pathological analysis of the subcellular localization of different isoforms of OCT4 will provide important clue on the diagnosis and prognosis of cervical cancer.

## Supporting Information

S1 Fig The subcellular localization of OCT4 in tumorspheres from primary tumors by IHC analysis. (TIF)

S2 Fig Immunohistochemical analysis of OCT4 localization in cervical cancer samples. Both OCT4 antibodies revealed nuclear location of OCT4A in human embryonic carcinoma cell line tera-1 (a, b). OCT4A antibody SC-5279 revealed nuclear staining of OCT4A in cervical cancer samples (c); but SC-8629 antibody revealed cytoplasmic staining of OCT4A. ×100 magnification.
(TIF)

S3 Fig Effect of OCT4B knockdown on cell viability and apoptosis. (A). SiHa cells were transfected with OCT4B-specific shRNA or scramble control shRNA. 72 h later, OCT4B mRNA expression was determined using quantitative real-time PCR. GAPDH was used as loading control. (B). OCT4B knockdown markedly inhibited cell proliferation. (C). OCT4B knockdown significantly increased cell apoptosis. *,*P*<0.05; **,*P*<0.01. Values were the mean±SD of three independent experiments.
(TIF)

## References

[1] Ferlay J, Shin HR, Bray F, Forman D, DM P GLOBOCAN 2008, Cancer Incidence and Mortality Worldwide: IARC CancerBase No. 10. Lyon, France: International Agency for Research on Cancer, 2010.

[2] Acladious NN, Sutton C, Mandal D, Hopkins R, Zaklama M, et al (2002) Persistent human papillomavirus infection and smoking increase risk of failure of treatment of cervical intraepithelial neoplasia (CIN). Int J Cancer 98: 435–439. 11920596 10.1002/ijc.10080

[3] Canavan TP, Doshi NR (2000) Cervical cancer. Am Fam Physician 61: 1369–1376. 10735343

[4] Reya T, Morrison SJ, Clarke MF, Weissman IL (2001) Stem cells, cancer, and cancer stem cells. Nature 414: 105–111. 11689955 10.1038/35102167

[5] Lopez J, Poitevin A, Mendoza-Martinez V, Perez-Plasencia C, Garcia-Carranca A (2012) Cancer-initiating cells derived from established cervical cell lines exhibit stem-cell markers and increased radioresistance. BMC Cancer 12: 48 10.1186/1471-2407-12-48 22284662 PMC3299592

[6] Cantz T, Key G, Bleidissel M, Gentile L, Han DW, et al (2008) Absence of OCT4 expression in somatic tumor cell lines. Stem Cells 26: 692–697. 18032701 10.1634/stemcells.2007-0657

[7] Liu D, Zhou P, Zhang L, Wu G, Zheng Y, et al (2011) Differential expression of Oct4 in HPV-positive and HPV-negative cervical cancer cells is not regulated by DNA methyltransferase 3A. Tumour Biol 32: 941–950. 10.1007/s13277-011-0196-z 21674242

[8] Ji J, Zheng PS (2010) Expression of Sox2 in human cervical carcinogenesis. Hum Pathol 41: 1438–1447. 10.1016/j.humpath.2009.11.021 20709360

[9] Gu TT, Liu SY, Zheng PS (2012) Cytoplasmic NANOG-positive stromal cells promote human cervical cancer progression. Am J Pathol 181: 652–661. 10.1016/j.ajpath.2012.04.008 22683467

[10] Scholer HR, Ruppert S, Suzuki N, Chowdhury K, Gruss P (1990) New type of POU domain in germ line-specific protein Oct-4. Nature 344: 435–439. 1690859 10.1038/344435a0

[11] Takeda J, Seino S, Bell GI (1992) Human Oct3 gene family: cDNA sequences, alternative splicing, gene organization, chromosomal location, and expression at low levels in adult tissues. Nucleic Acids Res 20: 4613–4620. 1408763 10.1093/nar/20.17.4613 PMC334192

[12] Atlasi Y, Mowla SJ, Ziaee SA, Gokhale PJ, Andrews PW (2008) OCT4 spliced variants are differentially expressed in human pluripotent and nonpluripotent cells. Stem Cells 26: 3068–3074. 10.1634/stemcells.2008-0530 18787205

[13] Wang X, Dai J (2010) Concise review: isoforms of OCT4 contribute to the confusing diversity in stem cell biology. Stem Cells 28: 885–893. 10.1002/stem.419 20333750 PMC2962909

[14] Lee J, Kim HK, Rho JY, Han YM, Kim J (2006) The human OCT-4 isoforms differ in their ability to confer self-renewal. J Biol Chem 281: 33554–33565. 16951404 10.1074/jbc.M603937200

[15] Gao Y, Wang X, Han J, Xiao Z, Chen B, et al (2010) The novel OCT4 spliced variant OCT4B1 can generate three protein isoforms by alternative splicing into OCT4B. J Genet Genomics 37: 461–465. 10.1016/S1673-8527(09)60065-5 20659710

[16] Tai MH, Chang CC, Kiupel M, Webster JD, Olson LK, et al (2005) Oct4 expression in adult human stem cells: evidence in support of the stem cell theory of carcinogenesis. Carcinogenesis 26: 495–502. 15513931 10.1093/carcin/bgh321

[17] Lengner CJ, Camargo FD, Hochedlinger K, Welstead GG, Zaidi S, et al (2007) Oct4 expression is not required for mouse somatic stem cell self-renewal. Cell Stem Cell 1: 403–415. 18159219 10.1016/j.stem.2007.07.020 PMC2151746

[18] Zangrossi S, Marabese M, Broggini M, Giordano R, D'Erasmo M, et al (2007) Oct-4 expression in adult human differentiated cells challenges its role as a pure stem cell marker. Stem Cells 25: 1675–1680. 17379765 10.1634/stemcells.2006-0611

[19] Liedtke S, Enczmann J, Waclawczyk S, Wernet P, Kogler G (2007) Oct4 and its pseudogenes confuse stem cell research. Cell Stem Cell 1: 364–366. 10.1016/j.stem.2007.09.003 18371374

[20] Liedtke S, Stephan M, Kogler G (2008) Oct4 expression revisited: potential pitfalls for data misinterpretation in stem cell research. Biol Chem 389: 845–850. 10.1515/BC.2008.098 18627312

[21] de Jong J, Looijenga LH (2006) Stem cell marker OCT3/4 in tumor biology and germ cell tumor diagnostics: history and future. Crit Rev Oncog 12: 171–203. 17425502 10.1615/critrevoncog.v12.i3-4.10

[22] Lengner CJ, Welstead GG, Jaenisch R (2008) The pluripotency regulator Oct4: a role in somatic stem cells? Cell Cycle 7: 725–728. 18239456 10.4161/cc.7.6.5573

[23] Cauffman G, Van de Velde H, Liebaers I, Van Steirteghem A (2005) Oct-4 mRNA and protein expression during human preimplantation development. Mol Hum Reprod 11: 173–181. 15695770 10.1093/molehr/gah155

[24] Cauffman G, Liebaers I, Van Steirteghem A, Van de Velde H (2006) POU5F1 isoforms show different expression patterns in human embryonic stem cells and preimplantation embryos. Stem Cells 24: 2685–2691. 16916925 10.1634/stemcells.2005-0611

[25] Mueller T, Luetzkendorf J, Nerger K, Schmoll HJ, Mueller LP (2009) Analysis of OCT4 expression in an extended panel of human tumor cell lines from multiple entities and in human mesenchymal stem cells. Cell Mol Life Sci 66: 495–503. 10.1007/s00018-008-8623-z 19023518 PMC11131475

[26] Papamichos SI, Kotoula V, Tarlatzis BC, Agorastos T, Papazisis K, et al (2009) OCT4B1 isoform: the novel OCT4 alternative spliced variant as a putative marker of stemness. Mol Hum Reprod 15: 269–270. 10.1093/molehr/gap018 19258399

[27] Folkman J (2002) Role of angiogenesis in tumor growth and metastasis. Semin Oncol 29: 15–18. 12516034 10.1053/sonc.2002.37263

[28] Amin R, Mishra L (2008) Liver stem cells and tgf-Beta in hepatic carcinogenesis. Gastrointest Cancer Res 2: S27–30. 19343145 PMC2661545

[29] Liu CG, Lu Y, Wang BB, Zhang YJ, Zhang RS, et al (2011) Clinical Implications of Stem Cell Gene Oct-4 Expression in Breast cancer. Ann Surg 253: 1165–1171. 10.1097/SLA.0b013e318214c54e 21394007

[30] Resende MFd, Chinen LTD, Vieira S, Jampietro J, Fonseca FPd, et al (2013) Prognostication of OCT4 isoform expression in prostate cancer. Tumor Biol. 10.1007/s13277-013-0817-9 23636800

[31] Rijlaarsdam MA, van Herk HA, Gillis AJ, Stoop H, Jenster G, et al (2011) Specific detection of OCT3/4 isoform A/B/B1 expression in solid (germ cell) tumours and cell lines: confirmation of OCT3/4 specificity for germ cell tumours. Br J Cancer 105: 854–863. 10.1038/bjc.2011.270 21847120 PMC3171004

[32] Wezel F, Pearson J, Kirkwood LA, Southgate J (2013) Differential expression of Oct4 variants and pseudogenes in normal urothelium and urothelial cancer. Am J Pathol 183: 1128–1136. 10.1016/j.ajpath.2013.06.025 23933063

[33] Kim RJ, Nam JS (2011) OCT4 Expression Enhances Features of Cancer Stem Cells in a Mouse Model of Breast Cancer. Lab Anim Res 27: 147–152. 10.5625/lar.2011.27.2.147 21826175 PMC3145994

[34] Cortes-Dericks L, Yazd EF, Mowla SJ, Schmid RA, Karoubi G (2013) Suppression of OCT4B enhances sensitivity of lung adenocarcinoma A549 cells to cisplatin via increased apoptosis. Anticancer Res 33: 5365–5373. 24324071

[35] Wang Yi-Dong, Cai N, Wu X-L, Cao H-Z, Xie L-L, et al (2013) OCT4 Promotes Tumorigenesis and Inhibits Apoptosis of Cervical Cancer Cells by miR-125b/BAK1 Pathway. Cell death & disease. 10.1038/cddis.2013.272 PMC3763434 23928699

[36] Monsef N, Soller M, Isaksson M, Abrahamsson PA, Panagopoulos I (2009) The expression of pluripotency marker Oct 3/4 in prostate cancer and benign prostate hyperplasia. Prostate 69: 909–916. 10.1002/pros.20934 19274762

[37] Wang X, Zhao Y, Xiao Z, Chen B, Wei Z, et al (2009) Alternative translation of OCT4 by an internal ribosome entry site and its novel function in stress response. Stem Cells 27: 1265–1275. 10.1002/stem.58 19489092

[38] Gao Y, Wei J, Han J, Wang X, Su G, et al (2012) The novel function of OCT4B isoform-265 in genotoxic stress. Stem Cells 30: 665–672. 10.1002/stem.1034 22247013

[39] Folkman J (2003) Angiogenesis and apoptosis. Semin Cancer Biol 13: 159–167. 12654259 10.1016/s1044-579x(02)00133-5

[40] Marks PA, Xu WS (2009) Histone deacetylase inhibitors: Potential in cancer therapy. J Cell Biochem 107: 600–608. 10.1002/jcb.22185 19459166 PMC2766855

[41] Thiery JP, Acloque H, Huang RY, Nieto MA (2009) Epithelial-mesenchymal transitions in development and disease. Cell 139: 871–890. 10.1016/j.cell.2009.11.007 19945376

