# Supplementary figures and images for: The Differential Expression of OCT4 Isoforms in Cervical Carcinoma
Source: PLoS One. 2015 Mar 27;10(3):e0118033. doi: 10.1371/journal.pone.0118033 (PMC4376746; doi:10.1371/journal.pone.0118033)

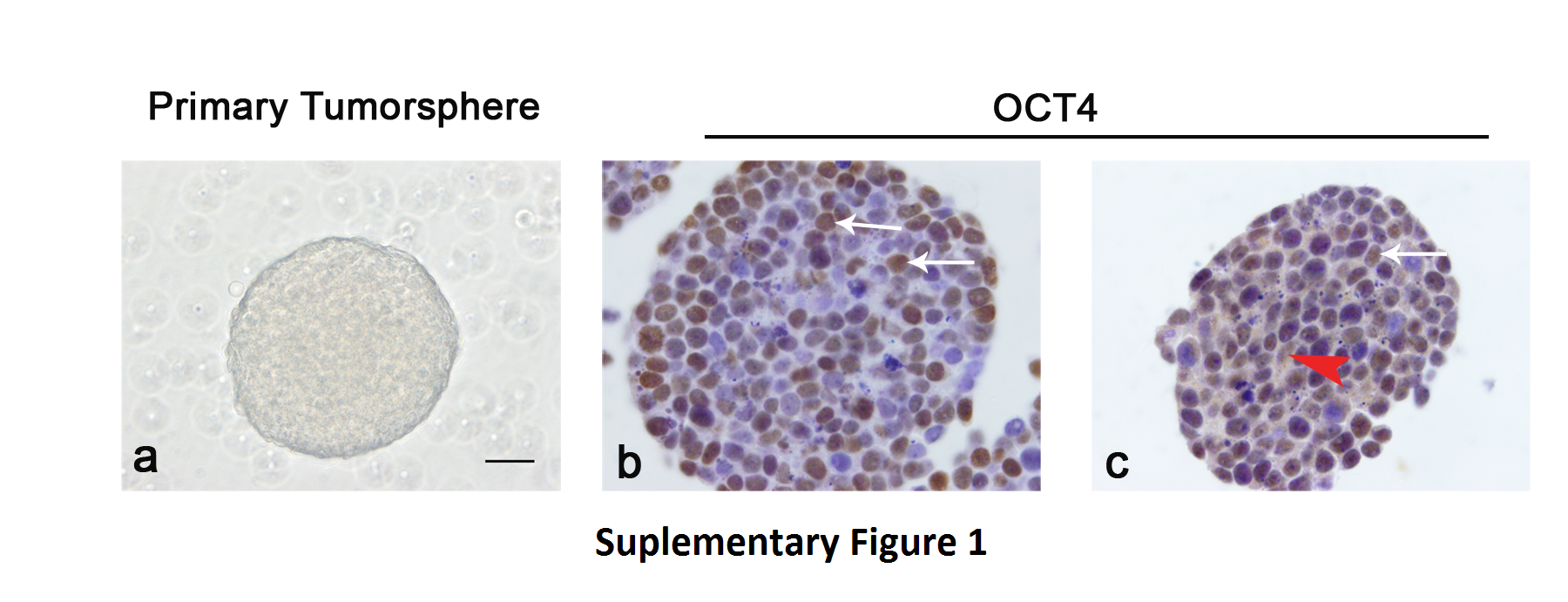

Supplement: S1 Fig — (TIF) [file pone.0118033.s001.tif]

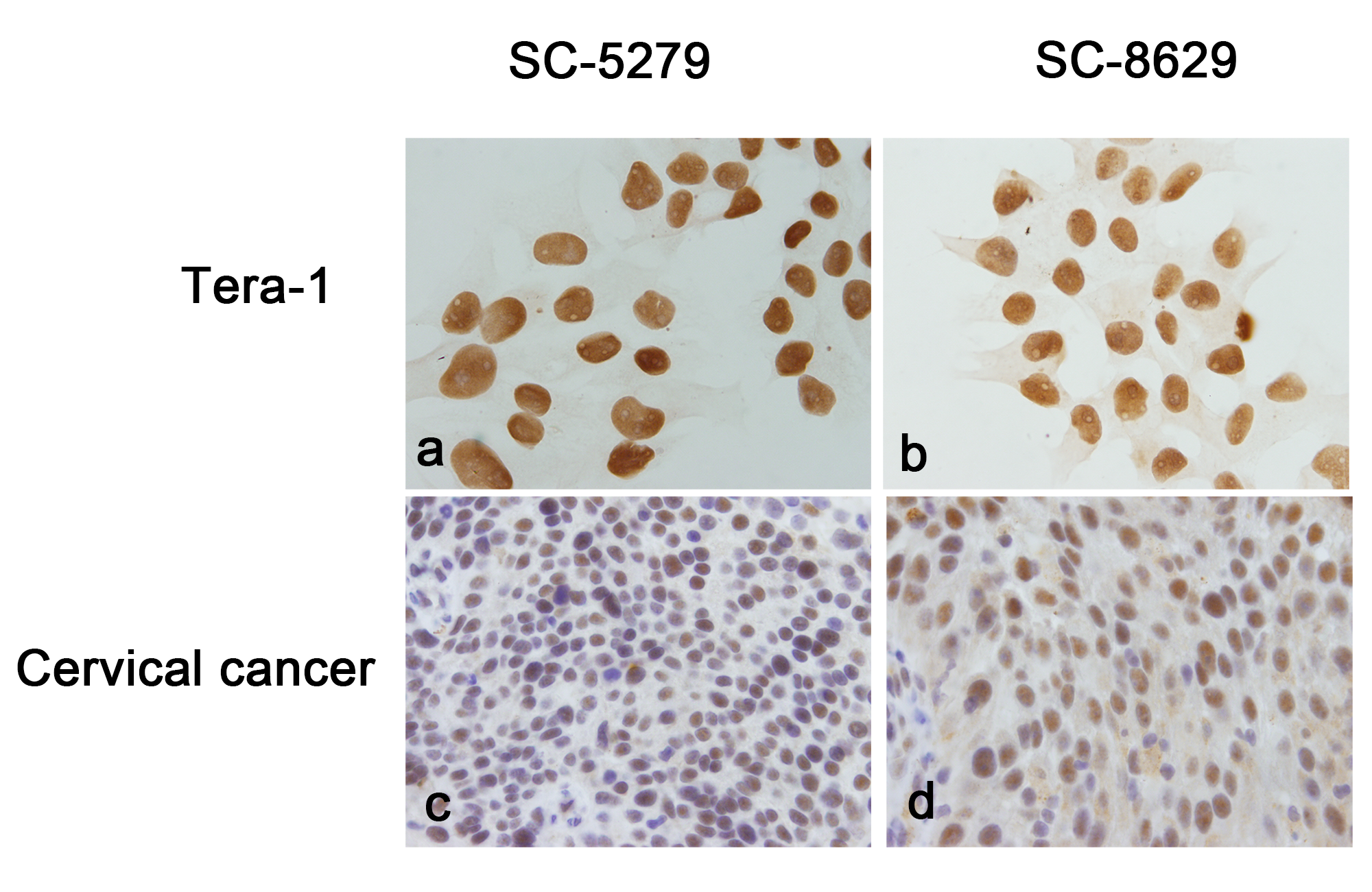

Supplement: S2 Fig — Both OCT4 antibodies revealed nuclear location of OCT4A in human embryonic carcinoma cell line tera-1 (a, b). OCT4A antibody SC-5279 revealed nuclear staining of OCT4A in cervical cancer samples (c); but SC-8629 antibody revealed cytoplasmic staining of OCT4A. ×100 magnification. (TIF) [file pone.0118033.s002.tif]

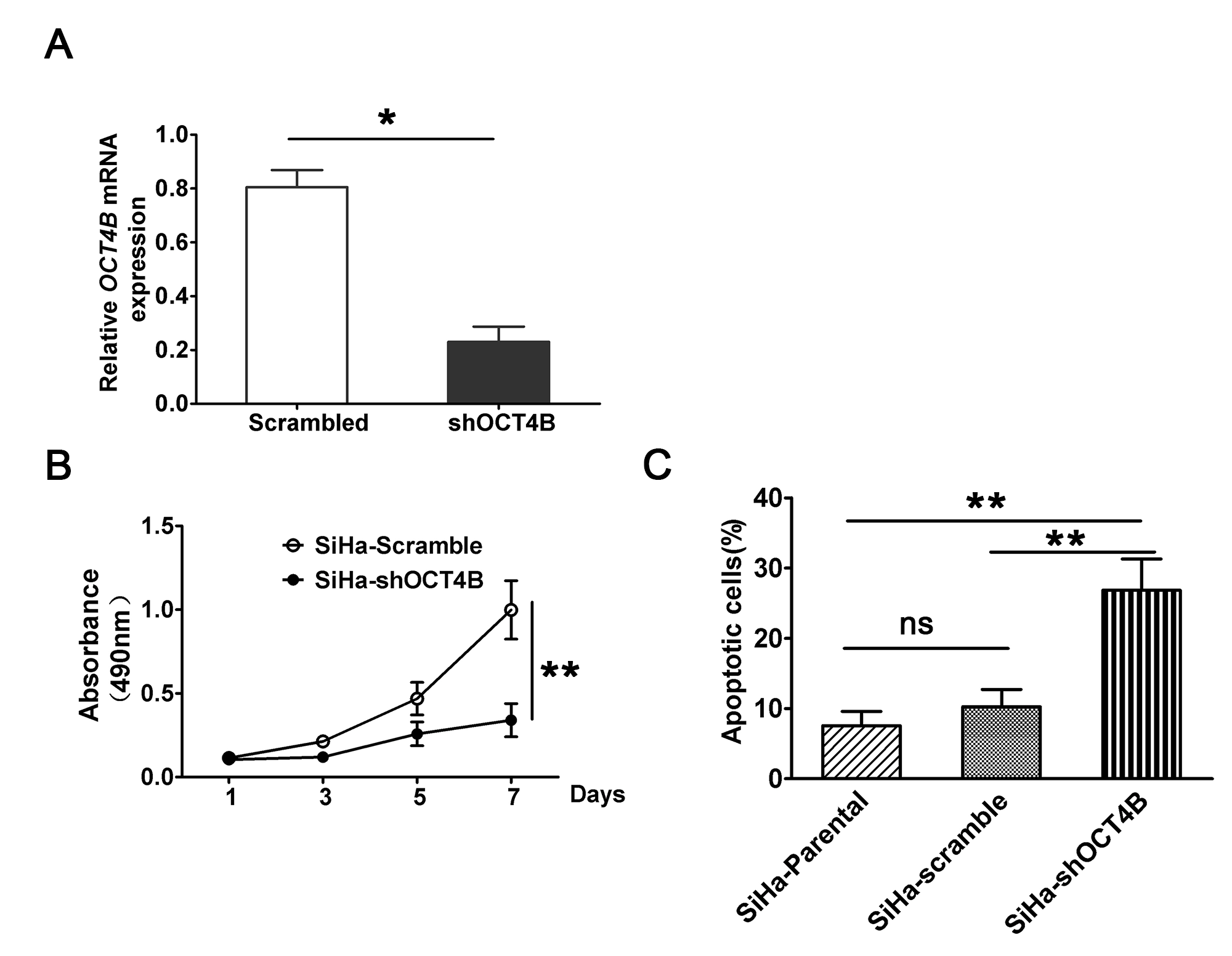

Supplement: S3 Fig — (A). SiHa cells were transfected with OCT4B-specific shRNA or scramble control shRNA. 72 h later, OCT4B mRNA expression was determined using quantitative real-time PCR. GAPDH was used as loading control. (B). OCT4B knockdown markedly inhibited cell proliferation. (C). OCT4B knockdown significantly increased cell apoptosis. *,P<0.05; **,P<0.01. Values were the mean±SD of three independent experiments. (TIF) [file pone.0118033.s003.tif]
